# Effect of percutaneous transluminal coronary angioplasty on QT dispersion and heart rate variability parameters

**Published:** 2009-08

**Authors:** Ali Aydinlar, Tunay Şentürk, Bülent Özdemir, Aysel Aydin Kaderli, Özlem Aydin

**Affiliations:** Department of Cardiology, Uludağ University School of Medicine, Görükle, Bursa, Turkey; Department of Cardiology, Uludağ University School of Medicine, Görükle, Bursa, Turkey; Department of Cardiology, Uludağ University School of Medicine, Görükle, Bursa, Turkey; Department of Cardiology, Uludağ University School of Medicine, Görükle, Bursa, Turkey; Department of Cardiology, Uludağ University School of Medicine, Görükle, Bursa, Turkey

## Abstract

**Background:**

The aim of the study was to analyse parameters reflecting the sympathovagal control of ventricular depolarisation and repolarisation [heart rate variability (HRV) and QT interval dispersion (QTd)] in patients undergoing elective percutaneous transluminal coronary angioplasty (PTCA), and determine whether HRV correlates with QT dispersion parameters.

**Methods:**

The study consisted of 26 consecutive patients (16 men, 10 women) with single-vessel coronary artery disease (CAD) who underwent elective coronary angioplasty. HRV analyses of all subjects were obtained with the time- and frequency-domain methods. For frequency-domain analysis, low-frequency HRV (LF), high-frequency HRV (HF) and the LF:HF ratio were measured. For time-domain analysis, standard deviations of the normal-to-normal QRS intervals (SDNN) and square roots of the mean squared differences of successive N–N intervals (rMSSD) were obtained. QT intervals were also corrected for heart rate using the Bazett’s formula, and the corrected QT interval dispersion (QTcd) was then calculated. All measurements (HRV parameters and QTcd) were made before and immediately after PTCA.

**Results:**

QTcd was significantly decreased after PTCA (52.2 ± 3.5 vs 42 ± 3.9 ms). SDNN (94.1 ± 22 vs 123.9 ± 35.2 ms), rMSSD (43.7 ± 20.1 vs 73.4 ± 14.5 ms) and HF (51.1 ± 48.8 vs 64.2 ± 28.6 ms^2^) were significantly higher after PTCA, whereas LF (142 ± 41.5 vs 157.2 ± 25.9 ms^2^) and the ratio of LF:HF (3.3 ± 1.9 vs 2.1 ± 1.2) were significantly decreased after PTCA. We observed a significant negative correlation after PTCA between QTcd and LF (*r* = −0.87, *p* = 0.01) and between QTcd and the ratio of LF:HF (*r* = −056, *p* < 0.05).

**Conclusion:**

Among the patients with CAD undergoing PTCA, QTcd significantly decreased after PTCA, and negatively correlated with LF, the parameter reflecting the sympathetic system.

## Summary

Impairment of autonomic cardiovascular regulation has been observed in patients with coronary artery disease (CAD).^1^ Sympathetic hyperactivity favours the genesis of life-threatening ventricular tachyarrhythmias,^2^ whereas vagal activation exerts an antifibrillatory effect.^3^ Percutaneous transluminal coronary angioplasty (PTCA) is widely used in patients with coronary artery disease. Balloon inflation followed by immediate reperfusion can cause myocardial ischaemia and can influence cardiac autonomic balance.

The QT interval on a 12-lead electrocardiogram (ECG) reflects ventricular repolarisation and the QT dispersion reflects dispersion of the ventricular refractoriness. Antzelevitch *et al*.^4^ reported that QT dispersions are heterogeneities of repolarisation time in the three-dimensional structure of the ventricular myocardium, which are secondary to regional differences in action potential during the activation time. Heart rate variability (HRV) has been shown to be a reliable non-invasive technique for the quantitative analysis of the activity of the two components of the autonomic nervous system.

The high-frequency band (HF: 0.15–0.40 Hz) in the energy spectrum of HRV is driven mainly by the respiratory rhythm and the energy in this band represents the parasympathetic modulation of the heart rate. The low-frequency component (LF: 0.04–0.15 Hz), however, is associated with vasomotor oscillations and reflects sympathetic modulation of the heart rate. The ratio of LF to HF (LF:HF) can be used as an index of sympathovagal balance modulating sinus node pacemaker activity.^5^ The parameters of HRV are determined by the rate of depolarisation, and the values of QT interval duration and its dispersion are reflected by repolarisation and its inhomogeneity.^5^ Significant correlations between HRV and QT in healthy persons have been reported recently by Ishida *et al*.^6^

There is, however, little information pertaining to the influence of elective PTCA on HRV and QT parameters. The purposes of this study were to measure changes in QTd and HRV before intracoronary balloon inflation, and immediately after balloon deflation, to determine whether HRV correlated with QT dispersion parameters, and whether the coronary artery vessel involved affected the QTd and HRV.

## Methods

A total of 26 patients with stable angina pectoris (16 men, 10 women, mean age 58.3 ± 17.1 years) were referred for elective PTCA. Single-vessel disease was defined as 70 to 99% diameter stenosis in the left anterior descending artery (LAD), the circumflex artery (LCx) or the dominant right coronary artery (RCA). The anatomical distribution of the coronary artery stenoses was as follows: the LAD in 12 patients, the LCx in six and the RCA in eight.

Systemic hypertension was considered to be present if the patient was taking an antihypertensive medication at the time of the hospital admission or if the systolic blood pressure was recorded as ≥ 140 mmHg and/or the diastolic blood pressure was ≥ 90 mmHg at least twice during examination on admission. A positive family history of premature CAD was defined as a first-degree relative who had documented CAD before the age of 55 years in males or 65 in females. For lipid analysis, samples were obtained on hospital admission, after an overnight fast. Those patients whose body mass indices were ≥ 30 kg/m^2^ were considered to be obese. Patients who had serum concentrations of total cholesterol (TC) ≥ 240 mg/dl, triglycerides ≥ 300 mg/dl, LDL-C ≥ 130 mg/dl, or high density lipoprotein-cholesterol (HDL-C) ≤ 40 mg/dl were considered to be hyperlipidaemic.

Patients excluded were those with myocardial infarction within the previous six months, second-, or third-degree atrioventricular conduction disturbances, atrial fibrillation or flutter, frequent (> 10/min) ventricular extrasystoles, sinus node disease, left ventricular hypertrophy, permanent ST changes on ECG, permanent cardiac pacemaker, abnormal serum electrolyte levels, ejection fraction < 45%, history of surgical revascularisation, congenital long-QT syndrome, an ECG with more than six missing leads, any disease state that could affect autonomic functioning, including diabetes mellitus, and patients taking drugs that modify the QT interval.

All patients were treated with standard medical therapy, including beta-blockers, aspirin, nitrate and heparin throughout the study period. Clopidogrel (75 mg/day) was started in addition to aspirin at least four days prior to the PTCA. The study protocol was approved by the Uludağ University Ethics Committee. Informed consent was obtained from each patient. The guidelines of the 2004 revision of Helsinki Declaration (1975) and good clinical practices were followed throughout the study.

All interventions were performed with conventional balloon PTCA. Cardiac catheterisation was done using the femoral arterial approach. Heparin was administered as a bolus dose of 10 000 U at the beginning of the procedure. The guide wire was advanced distally to the target lesion. The selection of balloon size was based on a visual estimation, using the guiding catheter for calibration. The balloon was placed at the site of the stenosis and inflated under continuous fluoroscopic observation. Balloon coronary occlusion was done according to the diameter of the coronary artery with a mean of 12.8 ± 2.2 atmospheres for 60 seconds. Only recordings of the first inflation were used in order to avoid ischaemic preconditioning. Bare metal and drug-eluting stents were used.

## QT dispersion measurement

The QT intervals and QTcd were measured manually from the standard ECGs available before and immediately after PTCA by two independent cardiologists. The 12-lead surface ECGs were recorded at a paper speed of 50 mm/s. The QT interval was measured from the onset of the QRS complex to the end of the T wave, defined as the return to the T–P isoelectric line. The QT was measured to the nadir of the curve between the T and U waves when the U wave was present. If the end of the T wave could not be reliably determined, such as in the case of a very low amplitude, QT measurements were not made and these leads were excluded from the analysis.

The QT interval was defined as the average of the QT intervals of three consecutive beats in each of the ECG leads. Dispersion of the QT interval was defined as the difference between the maximal and minimal QT interval measurements occurring in any of the 12 leads on a standard ECG. QT intervals were also corrected for heart rate using Bazett’s formula,^7^ and the corrected QT interval dispersion (QTcd) was then calculated.

## Heart rate variability

HRV was analysed as previously described, using data from Holter recordings (Synetec^™^ version 1.10, Ela medical, Montrouge, France), which were started on hospital admission. Immediately before balloon inflation, the event marker on the Holter recording was set. R–R data from Holter recordings were assessed using power spectral analysis; five minutes before balloon inflation, and five minutes after balloon deflation.

HRV was assessed in two ways: time- and frequency-domain analyses. Time-domain HRV indices: standard deviations of the normal-to-normal QRS intervals (SDNN) and square roots of the mean squared differences of successive N–N intervals (rMSSD) were calculated. Frequency domain HRV indices: the Fourier transform method was used for the spectral measurements, and the heart rate spectrum between 0.003 and 0.40 Hz was defined as total energy (ms^2^). This energy was divided into two components: low frequency (LF: 0.04–0.15 Hz) and high frequency (HF: 0.16–0.40 Hz).

## Statistical analysis

Statistical analysis was performed using SPSS for Windows 13.0 software. Categorical data are presented as absolute values and percentages, whereas continuous variables are summarised as mean values ± standard deviation. Because of the limited sample size and skewed distribution of the results, the Wilcoxon test was used to compare the mean values of the QT dispersion and HRV parameters recorded before and immediately after PTCA. Pearson’s correlation coefficients were analysed to examine the association between continuous parameters. Results were evaluated within the 95% confidence interval and *p*-values < 0.05 were accepted as significant.

## Results

Baseline patient characteristics are shown in Table 1. The patients’ mean age was 58.3 ± 17.1 years, 61.5% were male and 19.1% had diabetes mellitus. There were histories of smoking, hypertension, and hyperlipidaemia in 10 (38.5%), 11 (42.5%), and five patients (38.5%), respectively. Angiotensin-converting enzyme inhibitors, angiotensin II type 1 receptor antagonists and β-blockers were administered during hospitalisation in 18 (69.2%), six (23.1%), and 19 patients (73.1%), respectively.

**Table 1:** Patients’ Characteristics

No. of patients	26
Age (years)	58.3 ± 17.1
Gender (male:female)	16:10
Hypertension, *n* (%)	11 (42.3)
Hyperlipidaemia, *n* (%)	10 (38.5)
Family history of CAD, *n* (%)	11 (42.3)
Smoking, *n* (%)	10 (38.5)
Obesity, *n* (%)	4 (15.4)
EF (%)	58.9 ± 7.7
Medication	
Aspirin, *n* (%)	24 (92.3)
Beta-blockers, *n* (%)	19 (73.1)
Clopidogrel, *n* (%)	26 (100)
Nitrate, *n* (%)	20 (76.9)
ACE inhibitors, *n* (%)	18 (69.2)
ARB, *n* (%)	6 (23.1)
CCB, *n* (%)	7 (26.9)
Statin, *n* (%)	21 (80.7)

EF: ejection fraction, ACE: angiotensin converting enyzme, ARB: angiotensin receptor blocker, CCB: calcium channel blocker.

Each patient underwent successful and uncomplicated PTCA. The mean balloon inflation pressure was 12.8 ± 2.2 atmospheres. The mean balloon size was 28 ± 0.4 mm. The results of QTcd analysis are summarised in Table 2. The mean QTcd for all patients before the balloon inflation, and immediately after the deflation at five minutes were 52.2 ± 3.5 ms and 42 ± 3.9 ms, respectively (*p* = 0.03).

**Table 2:** QT Dispersion And Heart Rate Variability Parameters Before And After PCI

	*Before PTCA*	*After PTCA*	p*-value*
Heart rate	75.9 ± 13.6	74.8 ± 15.4	NS
QTcd (ms)	52.2 ± 3.5	42 ± 3.9	0.03
SDNN (ms)	94.1 ± 22	123.9 ± 35.2	0.01
rMSSD (ms)	43.7 ± 20.1	73.4 ± 14.5	0.01
LF (ms^2^)	142 ± 41.5	157.2 ± 25.9	0.041
HF(ms^2^)	51.1 ± 48.8	64.2 ± 28.6	0.02
LF:HF	3.3 ± 1.9	2.1 ± 1.2	0.032

PCI: percutaneous coronary intervention, QTcd: heart rate-corrected QT dispersion, SDNN: standard deviation of all normal R–R intervals, rMSSD: square root of the mean of the squared successive differences in R–R intervals, LF: low frequency, HF: high frequency, LF:HF ratio: low- to high-frequency ratio.

All measured parameters of HRV are presented in Table 3. SDNN (94.1 ± 22 vs 123.9 ± 35.2 ms), rMSSD (43.7 ± 20.1 vs 73.4 ± 14.5 ms) and HF (51.1 ± 48.8 vs 64.2 ± 28.6 ms^2^) were significantly higher after PTCA, whereas LF (142 ± 41.5 vs 157.2 ± 25.9 ms^2^) and the ratio of LF:HF (3.3 ± 1.9 vs 2.1 ± 1.2) were significantly lower after PTCA.

**Table 3:** Baseline Lesion And Procedural Characteristics

LAD, n (%)	12 (46.2)
LCx, n (%)	6 (23.1)
RCA, n (%)	8 (30.8)
No. of vessel disease	1.56 ± 0.58
Gensini scoring	21.1 ± 14.1
Stent type	
Bare metal	20
Drug eluting	6
Baloon pressure (atm)	12.8 ± 2.2
Inflation time (s)	60

LAD: left anterior descending artery, LCx: circumflex artery, RCA: right coronary artery, atm: atmospheres.

No significant correlations between HRV parameters and QTcd value were found before PTCA. After PTCA, we observed a significant negative correlation between QTcd and LF (*r* = −0.87, *p* = 0.01) and between QTcd and LF:HF (*r* = −056, *p* < 0.05).

QTcd and HRV changes were evaluated during coronary angioplasty according to the coronary artery involved. There was no correlation between these parameters and coronary artery involvement (*p* < 0.05). None of the 22 patients developed ventricular arrhythmias during or after the PTCA.

## Discussion

In this study, we specifically investigated changes in QTcd and HRV and their correlations in patients undergoing PTCA. The major findings of this study are (1) immediately after PTCA, QTcd decreased significantly in patients with CAD; (2) rMSSD and HF, which are the indicators of the parasympathetic nervous system activation, were increased, whereas LF, an indicator of sympathetic nervous activation, was decreased immediately after PTCA; and (3) a negative correlation was found between QTcd and LF, and the ratio of LF:HF immediately after PTCA.

HRV analysis is a safe and convenient method for the evaluation of the function of the autonomic nervous system in patients with various cardiovascular and non-cardiovascular disorders.^5^ Sympathovagal imbalance has been shown to be a strong and independent predictor of mortality in patients with myocardial infarction, heart failure, or diabetic neuropathy.^5^ HRV has been shown to be altered among patients with stable CAD and reduced even before the development of symptoms.^8^

Some investigators have reported changes in HRV during angioplasty in coronary artery disease.^5^,^9^ However, the conclusions of these studies are invalidated to some extent by the inclusion of patients with previous acute myocardial infarction, left heart failure and diabetes. Vagal activity is the major contributor to the HF component.^10^ The LF component is considered by some as a marker of sympathetic nervous activity and by others as the resultant of both sympathetic and vagal influences.^11^ Analysis of the LF:HF ratio rather than the single components is considered by many investigators to better reflect the activity of the sympathovagal balance.^5^ In the present study, the balance between sympathetic and vagal activity (ratio of LF:HF) significantly shifted toward vagal predominance immediately after balloon deflation.

Coronary occlusion may trigger several neural responses. Some are due to the stimulation of coronary mechanoceptors. Others arise from both ventricular mechano- and chemoreceptors that are activated mainly by occlusion and myocardial ischaemia.^12^ Stimulation of the coronary mechanoceptors of the left coronary artery may be elicited by an increase in coronary perfusion pressure as well as mechanical stretch, and may induce a reflex decrease in sympathetic drive.^13^ By contrast, reduced myocardial blood flow and ischaemia resulting from experimental occlusion of the left coronary artery may stimulate ventricular mechano- and chemoreceptors and increase activity in sympathetic efferent axons that move toward the heart.^14^

Reducing the inhibitory influences of vagal mechanisms or enhancing sympathetic activity might serve to prepare the cardiovascular system for the rapid variations in heart rate, cardiac output and redistribution of flow occurring during ischaemia.^15^ However, the neural sympathetic reflex seems to aggravate coronary ischaemia.^16^ Vagal activation exerts an antifibrillatory effect; sympathetic activation does the opposite.^3^ Our findings show that the components of HF, rMMSD and SDNN are significantly increased after PTCA. In our study, we observed significant reductions in the LF and LF:HF after PTCA, which points to sympathetic dominance of the control mechanisms in CAD. Contrary to our results, Airaksinen *et al*.^8^ suggested that impairment of the parasympathetic nervous function is common in coronary artery disease. Some investigators have suggested that increased sympathetic tone with elevated catecholamine levels may have direct effects on vascular smooth muscle cells, or that it may affect other factors promoting the progression of atherosclerosis.^17^,^18^

Niemela *et al*.^19^ showed that the relief of critical coronary obstruction by PTCA seemed to have no beneficial effect on vagal heart rate control. Airaksinen *et al*.^20^ showed that coronary occlusion causes immediate changes in HRV in more than one-third of patients with coronary artery disease. The direction of these initial HRV changes could not be predicted by the site of coronary occlusion. These observations were consistent with our study.

In order to standardise different studies investigating shortterm HRV, five-minute recordings of a stationary system are preferred.^5^ The spectral and time-domain HRV parameters were obtained from short recordings in our study, so we did not analyse HRV parameters during balloon inflation. Sustained ventricular tachycardia and ventricular fibrillation have been reported in 0.5 to 2% of patients undergoing coronary angioplasty.^21^ We did not observe ventricular arrhythmias during PTCA. We think that increasing parasympathetic activity and decreasing sympathetic activity after PTCA could reduce cardiac arrhythmias.

QT dispersion has been proposed as a marker of heterogeneous repolarisation and electrical instability.^4^ This has potential clinical value because heterogeneity in the recovery of ventricular excitability is presumed to increase the propensity for arrhythmic manifestations and arrhythmic death, especially in patients with previous myocardial infarction or history of CAD.^4^ Nowinski *et al*.^22^ observed that transient ischaemia during PTCA induced significant changes in ventricular repolarisation, especially during occlusion of the left anterior descending artery, and resulted in significant increase in QT interval dispersion. Aytemir *et al*.,^23^ during acute ischaemia provoked by angioplasty balloon in patients who had undergone PTCA, found higher values of QT interval dispersion when compared to the values before angioplasty. The results of other studies in patients undergoing PTCA confirm the ischaemic mechanism of QT dispersion changes.^23^,^24^

Our results are consistent with data reported by Yunus *et al*.,^24^ who showed that QT interval dispersion decreased after successful coronary artery revascularisation. Kajiyama *et al*.^25^ demonstrated that intracoronary balloon inflation did not modify the averaged QT intervals in patients with stable effort angina, but an increase in the dispersion of the QT interval during PTCA was associated with ventricular tachyarrhythmias. Our study differs from that of Kajiyama *et al*.25 in certain respects, because we analysed the QT interval before balloon inflation and after deflation to avoid the effect of ischaemic preconditioning on ventricular repolarisation.

We investigated changes in QTd before balloon inflation and immediately after deflation, according to the coronary artery vessel involved. Kılıç *et al.*^26^ showed that balloon inflation caused an increase in QTd but only in the LAD and RCA vessels. There was no statistically significant change in the lesions of the LAD, LCx or RCA before balloon inflation and immediately after deflation.

## Limitations

There are several important limitations to this study. The numbers in our patient population were limited. In order to eliminate the effects of lesions at non-PTCA sites on QT dispersion, only patients with single-vessel disease were included in our study. The patient population of our study consists of patients without a history of acute myocardial infarction, diabetes mellitus, and with preserved laft ventricular ejection fractions. Diabetes itself may be associated with depressed HRV due to diabetic autonomic neuropathy,^27^ and may result in reduced HRV inducing haemodynamic changes that involve adrenergic activation and vagal tone reduction. However, our patient group may not be representative of the overall patient population with coronary artery disease. From a methodological standpoint this may not have been the optimal approach.

Systemic catecholamine levels were not determined. Changes in respiratory rate are known to have an effect on HRV.5 We analysed HRV parameters before balloon inflation and immediately after deflation but we did not measure respiratory rate. Most the study patients were on similar anti-ischaemic therapy. In addition, because HRV is affected by various factors such as age of the patient,^28^ the use of angiotensin converting enzyme inhibitors^29^ and beta-blockers,^30^ these factors might have affected our results.

## Conclusion

The results of our study show that successful PTCA caused an improvement in the autonomic control of the heart immediately after PTCA and an improvement in HRV parameters, indicating parasympathetic nervous activation was negatively correlated with QTcd after PTCA. Successful PTCA may improve ventricular repolarisation abnormalities, and therefore it may reduce the incidence of ventricular arrhythmias. Our data were obtained from a small group of patients, and therefore need to be verified in a larger population of patients undergoing elective PTCA.
